# Time course gene expression profiling of yeast spore germination reveals a network of transcription factors orchestrating the global response

**DOI:** 10.1186/1471-2164-13-554

**Published:** 2012-10-15

**Authors:** Cecilia Geijer, Ivan Pirkov, Wanwipa Vongsangnak, Abraham Ericsson, Jens Nielsen, Marcus Krantz, Stefan Hohmann

**Affiliations:** 1 Department of Chemistry and Molecular Biology, University of Gothenburg, Box 462, Gothenburg, S-40530, Sweden; 2 Department of Chemical and Biological Engineering, Chalmers University of Technology, Gothenburg, S-412 96, Sweden; 3 Present address: Center for Systems Biology, Soochow University, Suzhou, 215006, China; 4 Present address: Theoretical Biophysics, Humboldt-Universität zu Berlin, Invalidenstr. 42, Berlin, 10115, Germany

**Keywords:** Sporulation, Germination, *Saccharomyces cerevisiae*

## Abstract

**Background:**

Spore germination of the yeast *Saccharomyces cerevisiae* is a multi-step developmental path on which dormant spores re-enter the mitotic cell cycle and resume vegetative growth. Upon addition of a fermentable carbon source and nutrients, the outer layers of the protective spore wall are locally degraded, the tightly packed spore gains volume and an elongated shape, and eventually the germinating spore re-enters the cell cycle. The regulatory pathways driving this process are still largely unknown. Here we characterize the global gene expression profiles of germinating spores and identify potential transcriptional regulators of this process with the aim to increase our understanding of the mechanisms that control the transition from cellular dormancy to proliferation.

**Results:**

Employing detailed gene expression time course data we have analysed the reprogramming of dormant spores during the transition to proliferation stimulated by a rich growth medium or pure glucose. Exit from dormancy results in rapid and global changes consisting of different sequential gene expression subprograms. The regulated genes reflect the transition towards glucose metabolism, the resumption of growth and the release of stress, similar to cells exiting a stationary growth phase. High resolution time course analysis during the onset of germination allowed us to identify a transient up-regulation of genes involved in protein folding and transport. We also identified a network of transcription factors that may be regulating the global response. While the expression outputs following stimulation by rich glucose medium or by glucose alone are qualitatively similar, the response to rich medium is stronger. Moreover, spores sense and react to amino acid starvation within the first 30 min after germination initiation, and this response can be linked to specific transcription factors.

**Conclusions:**

Resumption of growth in germinating spores is characterized by a highly synchronized temporal organisation of up- and down-regulated genes which reflects the metabolic reshaping of the quickening spores.

## Background

Cellular adaptation to environmental changes ensures fitness and survival of cells and organisms. Adaptation programs may strengthen cell walls in response to stress, switch the metabolic machinery upon starvation or trigger morphological differentiation during multicellular development and sexual reproduction. Entering and exiting G_0_, or quiescence, are yet further examples of cellular transitions, important for the health of all organisms. A greater understanding of the control mechanisms that establish, maintain and end quiescence is important for basal, medical and biotechnological research.

Budding yeast *Saccharomyces cerevisiae* responds to carbon and nutrient limitation either by entering stationary phase (haploids), or sporulation (diploids) [1,2]. Sporulation involves two distinct but tightly linked processes: meiosis and subsequent formation of four haploid spores, two each of mating type **a** and α, enclosed in an ascus sack (reviewed in [3]). The spores contain high levels of protective trehalose and are surrounded by multi-layered spore walls. These features enable them to withstand harsh environments and to survive long periods of dormancy [4-7]. Upon exposure to glucose and nutrients, spores exit dormancy through germination and resume growth. Spore germination is a multi-step process that includes local degradation of the thick spore wall, breakdown of storage carbohydrates, swelling and elongation of the tightly packed spore [8,9]. During germination, the spore gradually loses its spore characteristics such as resistance to chemicals, heat and UV light, and starts acquiring those of a vegetative cell [10,11]. If the germinating spore is in close proximity to cells of opposite mating type it will initiate mating within hours after germination, before re-entry into the mitotic cell cycle [11,12]. The mating process, which involves cell cycle arrest prior to bud-formation and DNA synthesis, at least partly explains the observation that DNA replication starts first a few hours into germination, compared to transcription and translation that initiate within minutes after addition of a glucose-containing rich growth medium [11,13-15]. The knowledge of the properties and activities of quiescent spores is relatively sparse. Deciphering the underlying control mechanisms of establishing, maintaining or exiting spore dormancy is important for better comprehension of all phases of the life cycle of yeast. Also the process of germination is poorly characterized and research in this field will broaden our understanding of cellular developmental processes.

In this study, we analysed the transcriptional reprogramming of germinating spores and show that exit from dormancy results in rapid and global changes consisting of sequential gene expression subprograms. We identified a complex candidate network of transcription factors (TFs) regulating this response, thereby extending our understanding of the global regulatory program during germination [11]. By comparison of the responses between rich growth medium and pure glucose, we find that although the expression outputs are qualitatively similar, the response to rich medium is stronger than the glucose response. Furthermore, spores treated with pure glucose sense and react on amino acid starvation within the first 30 min after germination onset, and this response can be linked to specific TFs. Taken together, these results highlight that the process of germination consists of specific and temporally distinct gene expression subprograms.

## Results

### Spore germination involves a global gene expression reprogramming that encompasses distinct subprograms

**Figure 1:**
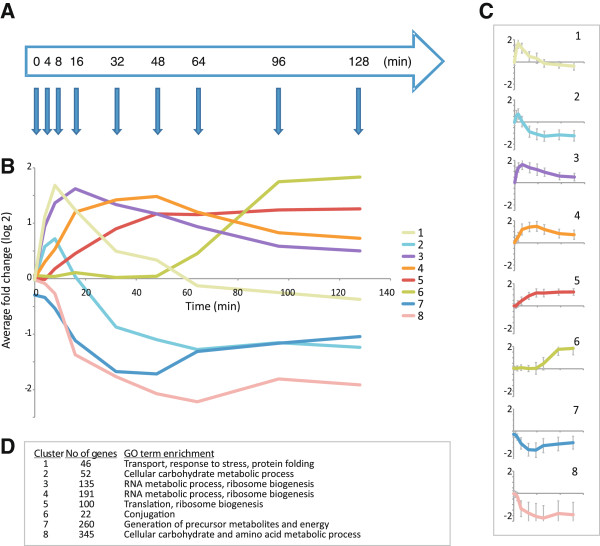
**The regulation of gene expression during germination encompasses a large number of genes and distinct regulatory programs.** (**A**) Experimental setup. Dormant spores were inoculated in pre-warmed YPD and allowed to germinate at 30°C under vigorous shaking. Samples were taken at the indicated time points (blue arrows), and mRNA expression levels throughout the time course were compared to that of dormant spores. (**B**) Genes that showed at least two fold altered expression in two or more consecutive time points during the germination process were subjected to K-means clustering and grouped in eight clusters. The right and lower panels display the (**C**) average expression profile for each cluster (error bars indicate standard deviation) and (**D**) the number of genes and enriched GO terms in each cluster.

We performed a time course microarray analysis to investigate the global response during germination (Figure 1A). The analysis showed that germination initiation is accompanied by a rapid and massive upregulation of gene expression: Hundreds of transcripts increase within minutes and 786 genes are sequentially upregulated (≥two-fold) during the first two hours of the germination process. To facilitate further analysis, we filtered for genes exhibiting at least a two-fold change in expression in two consecutive time points, which were considered as significantly regulated. This criterion yielded a set of 1,151 genes; 494 upregulated and 657 downregulated genes, as compared to dormant spores. K-means clustering analysis of these genes revealed eight clusters with different profiles and timings of up- or downregulation (Figure 1B & C). Interestingly, the clusters corresponded to distinct groups of genes with significantly enriched Gene Ontology (GO) terms (Figure 1D). Each cluster likely corresponds to one or several regulatory subprograms, which in turn is defined by distinct TF(s). K-means clustering of the complete set of genes is consistent with Figure 1, but the inclusion of ~5,000 genes that are not clearly responsive leads to dampened curves and three non-responsive clusters (and consequently merging of three pairs of the original clusters) (Additional file 1). Together, these results demonstrate that the exit from spore dormancy results in rapid and global expression changes with sequential onset of different subprograms.

### The regulatory subprograms are linked to distinct transcription factor sets

**Figure 2:**
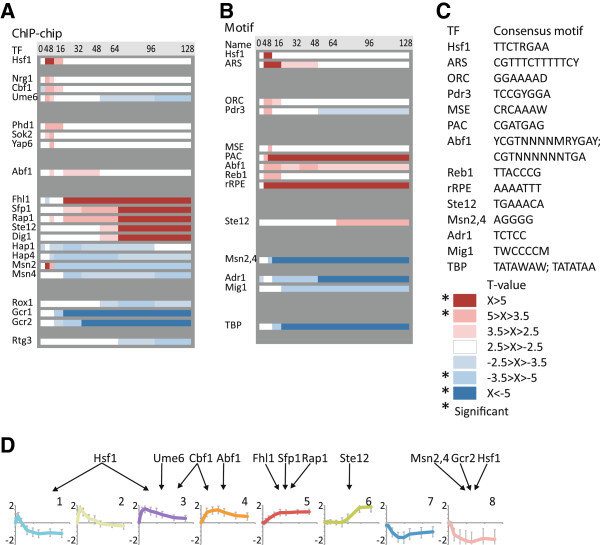
**Distinct transcription factor sets orchestrate different regulatory subprograms during germination.** T-profiler prediction of (**A**) TFs and (**B**) consensus motifs involved in the germination of yeast spores on YPD. The absolute T-values indicate the probability of the transcription factor/motif activity and the sign indicates if the expression of the target genes is increased (red) or decreased (blue). The response appears to have three major phases; an early transient peak (Hsf1, Nrg1, Phd1, etc.), a sustained adaptation (Fhl1, Hap1, Msn2, Gcr1, etc.) and, eventually, initiation of the mating response (Ste12, Dig1). (**C**) The TF activity predictions in (**B**) were based on the following consensus motifs for the respective TFs. (**D)** The TFs identified both in the global analysis in (**A**) and for the eight gene expression subprograms identified in Figure 1B .

We further aimed at identifying regulators of the global transcriptional germination program and its subprograms. The on-line-tool T-profiler [16] was used to predict the TFs responsible for the global germination program (Figure 2). We combined the results based on ChIP-chip data (Figure 2A) and consensus motifs (Figure 2B & C) to identify twenty-five potential global germination regulators. The collection of TFs reflects the shift to glucose metabolism and the consequential glucose repression (Rox1, Hap4, Adr1 and Mig1), the resumption of growth (Rap1, Sfp1 and Fhl1) and the release from stress (Msn2, Msn4) during germination. Interestingly, an early and transient activity of TFs was prominent, involving Ume6 and Cbf1 that share some highly upregulated target genes, as well as Hsf1 that regulates genes for chaperones involved in protein folding and refolding. Ume6 plays an essential role in sporulation by regulating early meiotic genes and was recently shown to be required also for germination [17]. We linked these TFs to the distinct subprograms identified in Figure 1 by analysing these clusters independently with T-profiler, relaxing the significance threshold, and taking the intersection between these results and the global germination TF set. Twelve of the twenty-five TFs were associated with distinct subprograms, with very limited overlap (Figure 2D). These TFs are largely consistent with the TFs identified for the clusters corresponding to the whole dataset in Additional file 1, further reinforcing the impression that the subprograms are regulated by distinct TF sets (Additional file 2).

### YPD and glucose results in qualitatively similar gene expression responses although YPD confer a stronger output than glucose

**Figure 3:**
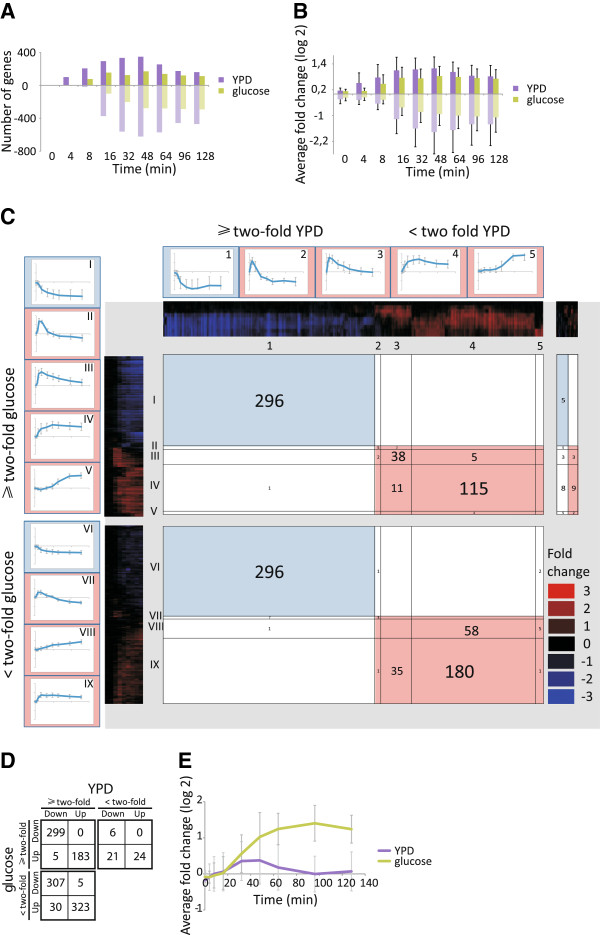
**The expression responses of spores to YPD and glucose are qualitatively similar although YPD confer a stronger output than glucose.** The gene expression program was compared between spores germinating in YPD and pure glucose media. (**A**) The number of core regulated genes changing at least two-fold up or down during the time course as positive and negative values, respectively. (**B**) The average fold change of the core regulated genes displayed as positive and negative values, respectively. (**C**) Two dimensional hierarchical clustering of the gene expression reprogramming during germination in YPD and glucose media. The x-axis represents the YPD cluster trees whereas the y-axis holds the glucose cluster trees. Three plots are separated by grey fields; genes two-fold changed in both the YPD and glucose data sets to the upper left, genes two-fold changed in glucose but not in YPD-induced germination to the upper right, genes two-fold changed in YPD but not in glucose induced germination to the lower left. Each cluster is subdivided into groups of genes with correlation factors ≥0.75 by thin black lines and the average expression profiles of groups of ≥ ten genes are framing the plots. The expression profiles of the genes that were up- or down regulated (≥two-fold) in glucose but not in YPD were heterogeneous in YPD (no clusters of ten or more genes with correlation factor ≥0.75) and are displayed in the upper right square. The colour panels along each axis indicates diminished expression of genes (blue) and stimulated genes (red), compared to dormant spores. Each gene occurs once on each axis and the number of genes (belonging to a cluster group of ≥10 genes with correlation factor ≥0.75) intersecting in each square is displayed. Also the genes in the upper right square are counted. The graphs displaying the average expression profiles are indexed with arabic and roman numerals for the YPD and glucose groups, respectively (error bars indicate standard deviation). The complete two dimensional hierarchical clustering with all genes included is depicted in Additional file 3. (**D**) The numerical summary of the two dimensional clustering, including all 1203 genes in the analysis, shows that the qualitative behaviour is very similar between the two germination conditions. However, as shown in (**E**), germination in glucose triggers a distinct subprogram encompassing genes in amino acid biosynthesis that is not induced in YPD,

The main trigger for spores to exit dormancy is a fermentable carbon source, which suffices to induce early germination events such as spore wall degradation and swelling [10,11]. To identify the impact of other components in rich growth medium, dormant spores were pulsed with pure glucose and samples were taken at the same intervals as the YPD treated spores for microarray analysis (Figure 1A), and the resulting expression profile was filtered as the YPD dataset. The genes significantly up- or down-regulated during germination in YPD and/or glucose define the core dataset used in the subsequent comparisons and consist of 1,203 genes. The response of spores to glucose was not as pronounced as to YPD, judged by both the number of significantly regulated genes and the average expression change of genes in the core dataset (Figure 3A, B). The average fold change was higher in YPD than in glucose even when the calculation was based on only those genes significantly regulated in both YPD and glucose, which excludes that this effect is solely due to regulation of additional subprograms in YPD that would bias the comparison (data not shown).

To further characterize common and unique aspects of the global gene expression profiles between spores germinating in YPD and glucose, two dimensional hierarchical clustering was performed (as described in materials and methods and elsewhere [18]). The core set of regulated genes was divided into two groups for each dataset based on expression change; genes altered at least two-fold and genes altered less than two-fold under that specific germination condition. These groups were clustered independently and plotted against each other in a two-dimensional matrix to determine the degree of correlation between the different spore responses. The matrix contains three groups of genes: those displaying a two-fold change in expression under both conditions, and those displaying a two-fold change in expression under either condition but not for the other (Figure 3C, Additional file 3). A simplified numerical analysis of the two dimensional cluster is depicted in Figure 3D. This presentation demonstrates the high degree of similarity in the gene expression response, and the numerical correlation between the responses was highly significant (p <10^-214^; chi-square test). We conclude that the vast majority of genes changed expression in the same direction, even though expression change for some genes passed the two-fold criterion in YPD but not glucose treatment. There is one noteworthy exception; 45 genes showed a two-fold or more up-regulation in glucose but not in YPD (Figure 3D). Almost half of these, 19 genes, encode proteins involved in amino acid biosynthetic processes, and their average expression profile was clearly different from that in YPD (Figure 3E). This strongly suggests that spores can sense and react on amino acid starvation within the first 30 min after germination onset.

### The differences between the transcriptional responses in YPD and glucose induced germination can be linked to specific TFs

**Figure 4:**
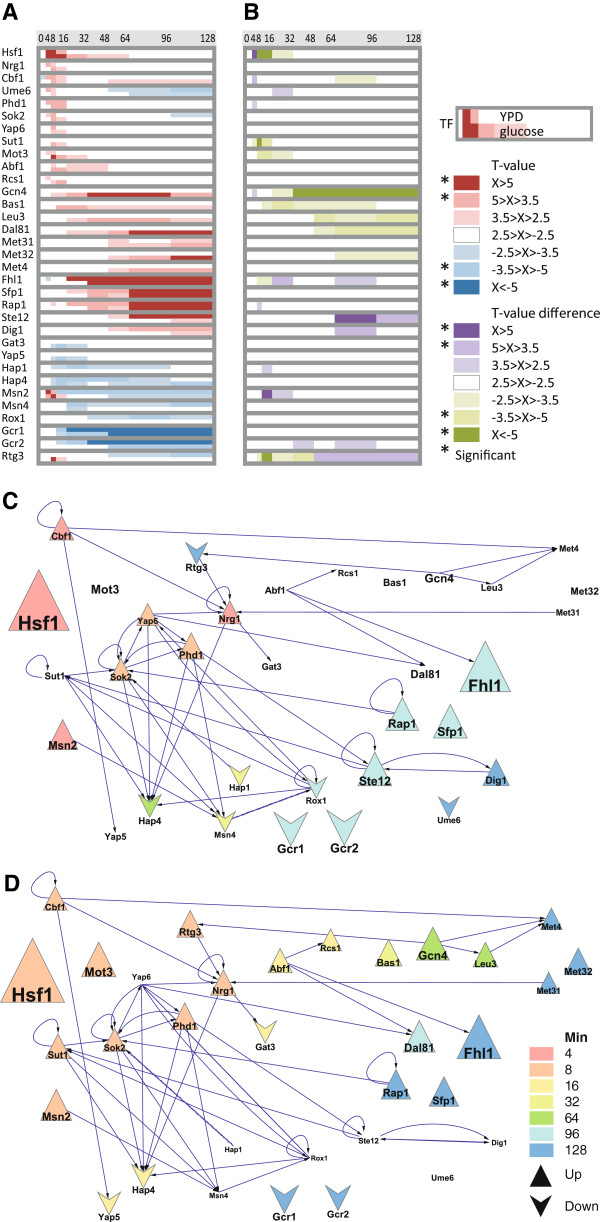
**Differences in expression regulation between YPD and glucose induced germination include the activity of TFs for amino acid biosynthesis.** (**A**) T-profiler prediction of TFs involved in the germination on YPD and glucose based on ChIP-chip results [20]. Colours indicate the relative TF activity (T-values) of YPD (upper row) and glucose (lower row) induced germination for each TF according to panel. (**B**) Differences in T-values for YPD and glucose germination highlight the differential induction of the amino acid biosynthetic subprogram (in glucose only) and timing of mating (faster in YPD). Differences of ±2.5 or more are indicated in purple (stronger in YPD) and green (stronger in glucose), and scaled according to the colour panel. Note that this colour indicates largest deviation from zero, not the highest value. White (and weak colours) indicate no significant difference. (**C**, **D**) The germination program is orchestrated by an interconnected network of TFs that are activated sequentially. TF node size indicates activity (T-profiler score), node colour indicates time of peak activity (see legend) and node shape indicates directionality (up or down). Edges show cross regulation within the TF network, i.e. reported TF binding to promoter of (other) TF [21]. Note that the timing is different in YPD (C) and glucose (D), and that different TFs are activated (missing nodes show no significant activity under that condition).

The similarity in gene expression pattern between germination in YPD and glucose is reflected in similar TF activity profiles. T-profiler analysis of the global germination program in glucose predicted eight additional TFs and the combined TF set of germination in YPD and glucose encompass thirty three TFs (Figure 4). The majority of these TFs show similar activity patterns under the two conditions (Figure 4A). To more clearly discern the condition specific differences, we plotted the difference in activity score over time with colour strength indicating absolute difference (Figure 4B). Part of these differences can be explained by the faster, stronger and perhaps more synchronous changes during germination in YPD, as exemplified by the Hsf1 peak and consistent with the gene expression data. However, the two conditions activate distinct subprograms, such as the amino acid biosynthetic genes induced during germination in glucose; involving Gcn4, Bas1, Leu3, Dal81, Met4, Met31, Met32 and Cbf1 that showed positive T-value profiles in glucose. Interestingly, Cbf1 seemed to have a dual role in germination, with high positive T-values at germination onset in both YPD and glucose, as well as a later function during germination specifically in glucose. Different subsets of Cbf1-target genes were upregulated in the two time windows and the later genes are targets of the Met4/Cbf1/Met28/Met31/Met32 activation complex that induce amino acid biosynthesis genes [19]. Hence, the TF activity profiles are highly similar during germination in YPD and glucose despite slight differences in timing and strength, and the activation of condition specific subprograms.

### The temporal ordering of the germination subprograms is consistent with the wiring of the TF network

To relate the temporal organisation to the candidate TF network structure, we extracted the TF sub-network corresponding to the thirty three TFs identified above from a global TF network [21]. Figure 4C and D display the cross regulation within the network as directed edges, and the peak activity score and timing of the peak for each individual TFs are indicated with node size and colour, respectively. This abstraction summarises the data with two exceptions; (i) Cbf1 has a double peak during germination in glucose, the second (of lower magnitude and hence not visualised) coincides with the amino acid biosynthetic activators, and (ii) Msn2 has an initial but very transient peak before falling down to a negative value, similar to Msn4, under both conditions. Note that the sequential order of activity is largely consistent with the network structure, including the synchronous peak of interconnected TFs such as Sok2 and Phd1, which appears to orchestrate a common, early gene expression subprogram, and that of the amino acid biosynthesis TFs Gcn4, Leu3 and Met4. We note that these two clusters overlap with the two out-of-phase sub-graphs proposed to define the cellular redox oscillations [21]. This reinforces the impression that a substantial part of the expression changes during germination reflect the metabolic evolution of the quickening spores rather than germination specific processes. It is tempting to speculate that the underlying regulation is related to the previously observed metabolic oscillations. In this scenario, the sharp transition from starvation to abundant energy leads to a rapid and highly synchronised initiation of metabolism followed by a synchronised metabolic evolution in the spores, which would allow the metabolic phases to be observed on the population level. However, the germination process also includes specific subprograms, such as the activation of Hsf1 and the initiation of mating. Taken together, the predicted TF activities display a temporal organisation which is consistent with the cross regulation within the TF network and which may largely reflect the metabolic evolution of the quickening spores.

### The spore germination gene expression program overlaps between yeast strains but not with the genes reported to be required for germination

**Figure 5:**
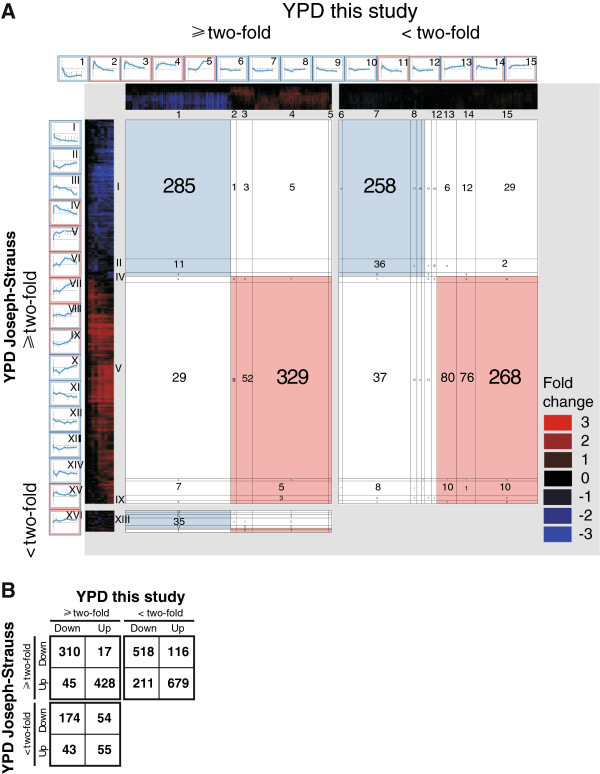
**Comparison to a previous study confirms a core germination program and pinpoints novel regulated gene groups during germination onset.** (**A**) Two-dimensional hierarchical clustering of the germination program observed here (x-axis) and reported previously (y-axis); both in YPD [11]. As in Figure 3, three plots are separated by grey fields; genes two-fold changed in both data sets to the upper left, genes two-fold changed in the Joseph-Strauss dataset but not in our data to the upper right, genes two-fold changed in our data but not in the Joseph-Strauss dataset to the lower left. Each cluster is subdivided into groups of genes with correlation factors ≥0.75 by thin black lines and the average expression profiles of groups of ≥ ten genes are framing the plots. The colour panels along each axis indicates diminished expression of genes (blue) and stimulated genes (red), compared to dormant spores. Each gene occurs once on each axis and the number of genes (belonging to a cluster group of ≥10 genes with correlation factor ≥0.75) intersecting in each square is displayed. The graphs with average expression profiles are indexed with arabic and roman numerals for the groups in our data and the Joseph-Strauss data, respectively. The complete two dimensional hierarchical clustering with all genes included is depicted in Additional file 4. (**B**) Numerical representation including all 2650 genes in the analysis.

We proceeded to compare our results to a previous transcriptome analysis of germination of SK1 spores [11]. Two dimensional hierarchical clustering of genes exhibiting at least a two-fold change in expression in two consecutive time points shows that these previous results are largely consistent with our findings, despite the use of an auxotrophic strain and differences in sporulation procedures, number of replicates and choice of reference RNA (Figure 5A, Additional file 3). In fact, the simplified numerical analysis shows that their set encompasses 71% of the genes identified here during germination in YPD (Figure 5B). However, we only confirm 34% of the genes reported in this previous dataset. This is likely due to more stringent inclusion criteria (triplicates), as the gene clusters we report here are more coherent; we report fewer and relatively larger gene clusters (5 vs. 9; 20% vs. 11% per cluster), as determined by separation at correlations coefficients <0.75 (see methods for details). Furthermore, the data presented here have a higher time resolution during the onset of germination, and a unique feature in this study is the rapid but transient upregulated genes (Figure 1B). In contrast to the large consistency between these two datasets, there is no significant overlap with the 158 germination deficient mutants identified by Deutschbauer et al. [22], although there is a tendency towards underrepresentation among the genes upregulated during germination (p = 0.1, data not shown). It has been observed and discussed before that the overlap between mutant and gene expression data when studying yeast responses show little or no overlap [23]. To summarize, the data presented here help narrowing down the germination program substantially and identify previously uncharacterised subprograms in the very onset of the germination process, which display no significant overlap with germination defective mutants.

### The gene expression programs of germination and stationary phase exit exhibit common and unique features

**Figure 6:**
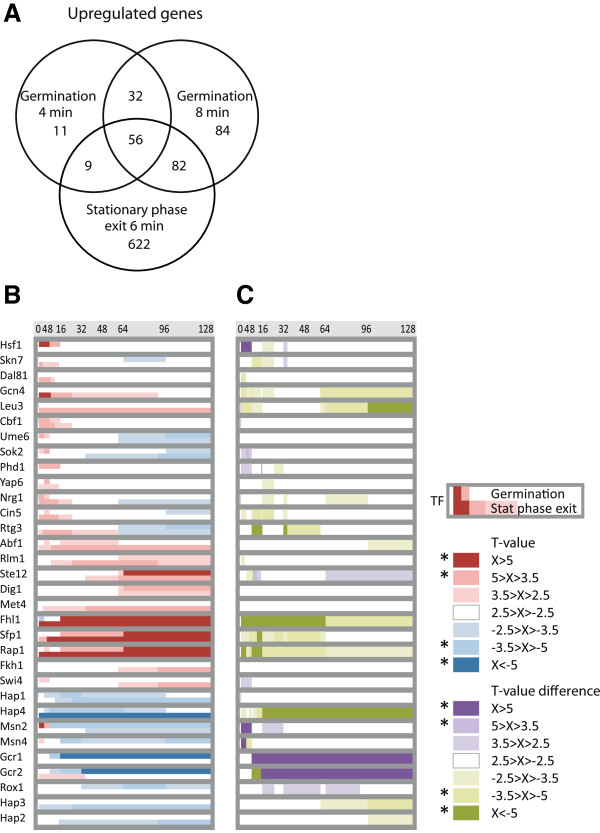
**The gene expression program during germination and stationary phase exit largely overlap.** (**A)** Venn diagram visualizing the overlap of genes ≥ two-fold upregulated within the first minutes after addition of rich growth medium (YPD) to stationary phase cells [24] and spores (germination in YPD; this study). These time points show the overlap of upregulated genes during the first minutes upon dormancy exit. (**B**) T-profiler analysis of the first two hours of YPD-induced dormancy exit displayed as T-scores for each TF during germination (upper line) and stationary phase exit (lower line), and as (**C**) differential display showing the difference in T-score when either germination (purple) or stationary phase exit (green) is stronger. The two gene expression programs largely overlap (all white fields) but show some distinct characteristics.

Several lines of evidence suggest that a large portion of the germination program reflects the metabolic evolution of the quickening spores rather than germination specific functions. To pursue this connection in more detail, we compared the germination program to the transcriptional changes during stationary phase exit [24]. We started with a detailed analysis of the early time points (4–8 min) in our YPD dataset which revealed a novel transient peak of up-regulated genes including transcripts for protein folding, transporters and transcriptional regulators. A numerical comparison showed that more than half of the genes (147 out of 274) that we found to be at least two-fold upregulated at 4 and 8 minutes during germination in YPD, were also upregulated 6 min after exit from stationary phase (Figure 6A).

Further, a T-profiler analysis of stationary phase exit and comparison with YPD-induced germination revealed that the rapid and transient upregulation of many genes seen during germination was also prominent during stationary phase exit (Figure 6B, C). Whereas Cbf1 activity appears in both exit programs, Hsf1 seems germination specific. Several discrepancies may be due to differences in the initial states of spores and stationary phase cells, such as the faster upregulation of ribosomal biosynthesis and increased downregulation of respiration in the latter (Fhl1/Sfp1/Rap1 and Hap2/Hap3/Hap4). Others may reflect strain differences, such as the induction of amino acid biosynthetic genes in the auxotrophic strain during stationary phase exit (Gcn4/Leu3). Hence, the two conditions differ in subprogram composition, timing and amplitude, but also share a number of changes towards growth and differentiation, such as the massive induction of ribosomal biosynthesis and the induction of mating (Ste12).

## Discussion

The ability to exit and re-enter the cell division cycle in response to altered nutrient conditions is of paramount importance for cellular viability and cooperation. When starved, diploid yeast cells enter the sexual cycle by sporulation, while haploid cells arrest in a stationary phase. Cells in these two quiescent stages share many properties; they do not proliferate, their metabolic, transcriptional and translational rates are low, and they are surrounded by a thickened cell wall that provides resistance to harsh environments [1]. Consistently, their modes of re-entry into active division share many features, including initiation by a fermentable carbon source, acquisition of increased sensitivity to cell wall degrading enzymes and heat, and they rely on similar gene expression programs as described here and previously [11,24]. It has been reported for stationary phase cells that RNA Polymerase II is positioned upstream of genes rapidly expressed upon addition of nutrients and glucose [24], and we find that many of the same genes are highly responsive during onset of spore germination. This indicates that a similar positioning may exist in spores, although further experimental evidence remains to be provided.

Further, we identified a common early and transient peak of transcription, including genes encoding transporters and transcriptional regulators that presumably are required for setting up proliferation after spore dormancy [24]. We also see that glucose repression is quickly turned on and that mRNAs for gluconeogenesis, the TCA cycle and the carboxylate cycle are rapidly and strongly down-regulated both during germination and stationary phase exit (Hap 2–4, Rox1; Figure 5) [11,24]. The identification of these subprograms also during germination advances our understanding of the germination program and further bridges the differences between re-entry from the different quiescent stages.

There are also discrepancies between dormancy exit of spores and stationary cells. In our hands, germination is associated with down-regulation of Gcr1/Gcr2-regulated glycolytic mRNAs, which does not occur during quickening from stationary phase. This is counterintuitive as resumption of growth in glucose containing medium requires glycolysis. We can only speculate about the possible purpose of this. To germinate quickly is crucial in the competition with other micro-organisms for nutrients and colonization of the micro environment; perhaps the spores contain high levels of these glycolytic mRNAs for translation immediately upon germination signals as mRNAs already present in the dormant spores most likely is the first choice for translation upon germination initiation [25]. It is also possible that the mRNAs of glycolytic genes, if bound to ribosomes or accumulated in P-bodies, are protected from degradation in the dormant spore and therefore enriched. Whether these expression changes have any impact on the glycolytic protein levels during germinating still needs to be determined. We also see that Hsf1 activation of genes associated with protein folding and refolding is more prominent during germination than stationary phase exit. The dormant spore contains high levels of trehalose that serves a protective role for proteins [5,26,27]. This trehalose is rapidly mobilised during germination and the Hsf1 mediated response may be required to protect or refold proteins during this transition [28,29]. Consistently, trehalose accumulates at high temperatures [29,30], while protein levels of chaperone Hsp104 increase only upon heat stress relief (Youlian Goulev, personal communication). Stationary phase cells also accumulate trehalose [1], and we note that genes implicated in protein (re)folding are up-regulated also during exit from stationary phase. The absence here of a significant peak of Hsf1 activation could reflect that these cells accumulate relatively little trehalose, are less dehydrated or that the first time point examined, six minutes into the quickening process, is too late to capture the Hsf1 peak. Hence, most apparent discrepancies between the two quiescent states may be quantitative rather than qualitative.

We further characterise the spore quickening by examining the effect of re-entry of media on the gene expression program. Rich growth medium with glucose has proved to be a more powerful germinant than glucose alone when it comes to acquisition of zymolyase sensitivity and trehalose breakdown [10,28]. This holds true also at the level of gene expression; although the transcriptional outputs are qualitatively very similar, YPD induces a stronger, faster and/or more synchronised response than pure glucose. These results support the idea that spores sense not only glucose but also the presence of nutrients and growth factors in the environment. In particular, the presence of amino acids appears essential for the rapid and massive induction of ribosomal genes that we observe exclusively in YPD [11]. This implicates the TOR pathway in the quickening process but it remains unclear if glucose is the initial trigger that enables other nutrients to be sensed, or if energy and other nutrients are sensed simultaneously. We note that in glucose-treated spores, TF activity and genes for amino acid biosynthesis are induced within half an hour, clearly showing that the limitation of amino acids is sensed early during the germination process. The ribosomal protein genes are induced within a similar time window, suggesting that the delay in ribosomal up-regulation reflects the delayed amino acid supply in glucose The TFs for amino acid biogenesis displayed rapid and sustained induction also during YPD-induced stationary phase exit (Gcn4 and Leu3; Figure 5), possibly reflecting differences between the auxotrophic BY4741 used for stationary phase exit and the prototrophic Y55 strain used in this study. However, this is not reflected in a delayed induction of the ribosomal protein genes showing that they are regulated by distinct mechanisms and/or cues.

Finally, we resolve the gene expression profile in temporally distinct subprograms that are orchestrated by interconnected TFs. The data set we present here has a higher initial time resolution than previous studies, more stringent evaluation criteria, less noise and hence more coherent clusters, and benefit from the increased resolution gained by two-dimensional clustering. This allows us to analyse the temporal and compositional organisation of the germination gene expression program. As shown in Figure 4, the candidate TF network driving the quickening is highly interconnected and the temporal organisation of the response reflects the interconnections in this network and the activities of interconnected TFs are highly synchronous. In particular, we see an early peak of the Sok2-Yap6-Phd1 cluster, which has been implicated in the metabolic and respiratory oscillations [21]. This is paralleled by a strong activity in Hsf1 as discussed above. The Sok2-Yap6-Phd1 peak is followed by rapid and sustained induction of ribosomal genes in YPD (Fhl1, Sfp1, Rap1) and biosynthetic genes (Gcn4, Bas1, Leu3, Dal81) in glucose where ribosomal protein genes do not peak until after full induction of the biosynthetic program (Figure 4). Interestingly, the Hsf1 peak is sustained until the ribosomal induction, which may suggest that its down-regulation is dependent on amino acid and/or protein synthesis. This delayed response in glucose is also reflected in a slower mating response, again potentially reflecting a need for protein synthesis in the acquisition of sexual (haploid) identity. Finally, germination in glucose leads to a second biosynthetic peak (Met31-Met32-Met4-Cbf1) that is absent in YPD. Also this cluster has been implicated in metabolic oscillations and constitutes a key regulator of the biosynthetic program peaking in the oxidative phase [21]. The absence of this peak in YPD could be due to the abundant amino acids in rich media or the entry into a different subprogram: Mating. Taken together, the transition from quiescence to growth primarily includes metabolic changes and key regulators of oscillatory metabolism appear to strongly influence also germination, which may reflect a highly synchronised return to active metabolism. We find the lack of germination specific targets noteworthy and conclude that this highly complex program is primarily a metabolic adaptation that adapts its subprogram composition to the exact environmental conditions of the quickening spores.

## Conclusions

In conclusion, we have determined the gene expression response of dormant spores to YPD and pure glucose, identified distinct expression subprograms, and linked them to distinct TFs. We found strong similarities between quickening of spores on YPD and glucose, and to quickening from stationary phase. Resumption of active metabolism coincides with a transcriptional response with clearly distinct subprograms that reflect each nutritional condition. Interestingly, the program does not encompass any clear germination specific genes, and the process can be explained by a combination of metabolic programs including trehalose mobilisation, glucose repression, amino acid and protein biosynthesis, and mating. Future challenges in the field of yeast germination include deciphering the translational and metabolic responses of germinating spores, and to identify proteins and pathways essential for the process of exiting dormancy.

## Methods

### Sporulation and germination conditions

Diploid cells of Y55 (*HO gal3 MALI SUCI*) strain background [31] were pre-cultured in rich growth medium (YPD: Yeast extract 1%, Peptone 2% Glucose 2%), transferred to pre-sporulation medium YPAc (Yeast extract 1%, Peptone 2%, 1% Potassium Acetate) and grown for ~14 h at 30°C under vigorous shaking. Cells were then transferred to sporulation medium (1% Potassium Acetate) and allowed to sporulate for 3–4 days at 30°C under vigorous shaking. Spore purification was performed with the use of zymolyase as described previously [10]. Finally, spores were washed from cell debris of non-sporulated vegetative cells and asci sacks by centrifugation. Spores were sonicated (3 x 15 sec, 10 μλ) to ensure freed spores, and kept at 4°C in 0.5% Triton X until used. Prior to germination, spores were incubated at 30°C for 30 min, and germination was initiated by addition of pre-warmed YPD or 2% glucose.

### Transcriptome analysis

Samples for RNA isolation were taken in three biological replicates at nine different time points (0, 4, 8, 16, 32, 48, 64, 96, 128 min) after addition of YPD or glucose 2%. For RNA extractions, 10 ml sample volumes were taken at each time point and transferred to 40 ml ice-cold 0,5% TritonX-100 in 50 ml falcon tubes, left on ice for a few minutes and then centrifuged briefly to collect the cells (1 min at 4000 rpm, 2°C). The pellet was immediately frozen in dry-ice. Total RNA was extracted as described previously [32]. 16 μg of total RNA were primed with 3 μg of random hexamer (Invitrogen) and 3 μg of anchored oligo(dT)_20_ primer (ABgene) and labelled in a reverse transcription reaction with Cy3-dUTP or Cy5-dUTP (Amersham Pharmacia Biotech) in a total volume of 30 μl, following standard protocols (http://.cmgm.stanford.edu/pbrown) [33]. Labelled cDNA was cleaned, combined, vacuum-dried, and resuspended in 80 μl of DIGeasy hybridization buffer (Roche Diagnostics). The hybridization mix was placed at 100°C for 2 min and then at 37°C for 30 min. Before hybridization, the microarray chip (Yeast 6.4 KB, double-spotted array containing 6240 yeast ORFs, http://microarrays.ca) was prehybridized with 1% BSA in DIGeasy hybridization buffer at 42°C for 1 h. Hybridization was performed at 42°C for 12–18 h. The slides were scanned on a GenePix 4000B scanner and quantized using the software GenePix Pro 4.0 resulting in data for 13056 spots, two spots represented one gene. RNA from dormant spores was used as reference RNA for all microarrays.

For microarray data analysis, normalization was performed by a correction for dye-bias and intensity dependent trends. The normalization method was chosen by fitting a robust line (loess line) on the M-values and then corrected each value according to the value of the line. Statistical data analysis was carried out to obtain significantly different gene expression by using a moderated t-test analysis in R and Limma package [34]. Empirical Bayesian statistics were used to moderate the standard errors within each gene and Benjamini-Hochberg method [35] was applied for correction of multiple testing. Each comparison consists of three (two for glucose 0 min) independent experiments.

Transcription profiles of germination and stationary phase comparative datasets were collected from databases NCBIs Gene Expression Omnibus (Series accession number GSE7393), and The ArrayExpress Archive (Experiment E-UMCU-12), respectively. Normalized log2 values were subjected to transformation so that all genes were 0 at 0 min time points.

### Availability of supporting data

MIAME-compliant microarray data has been deposited in the microarray database GEO (http://www.ncbi.nlm.nih.gov/geo/) with the accession number GSE29960.

### Filtering and clustering

The gene expression profiles were filtered to select genes with a reliable expression change by applying the criterion of a two-fold change (in the same direction) in expression level in two or more consecutive time points. K-means and hierarchical clustering was performed and visualized using Cluster and TreeView (http://.rana.lbl.gov/EisenSoftware.htm) [36]. Two dimensional hierarchical clustering was performed as described elsewhere [18]. Briefly, genes passing the filter in one or both conditions made up the core regulated dataset. Each germination condition was handled independently and two cluster analyses were performed: (i) clustering of genes displaying a two-fold change in expression level following addition of that particular germinant (ii) clustering of genes displaying a two-fold change in expression level in the second germinant. Comparison was done by plotting the clusters against each other. Average expression profiles was plotted of groups with 10 or more genes with correlation factor of ≥0.75. For statistical analysis, chi-square test was performed. GO term enrichment analysis was done using Yeast GO slim (process) at the SGD homepage (http://www.yeastgenome.org).

### Transcription factor identification

T-profiler [16] was used to identify TFs and upstream consensus motifs. This online tool (http://www.t-profiler.org) compares the mean expression ratios of groups of genes, and all genes within each group contribute to the evaluation of statistical significance, not just those genes that are judged to be differentially expressed. Two statistical parameters are utilized; (i) a *t*-value measuring the up-regulation (*t* > 0) or down-regulation (*t* < 0) in units of the standard error of the difference and (ii) a Student *t* test derived E-value that reflects the degree of difference in the mean log_2_-transformed expression ratio of a pre-defined group of genes and the mean for the rest of the genome. An E-value of <0.05 is considered as a statistically significant difference in gene expression. Only TFs and motifs with T-values ≥3.5 or ≤ −3.5 (in one or more time points) in one or both conditions compared are included in the analyses. Most but not all of these TFs have statistically significant E-values. Where several T-values for a transcription factor are identified, the highest absolute T-value at each time point is used. For TF identification in k-means clusters, the time point with the largest change compared to previous time point for each cluster is analysed. Only those TFs identified also for the whole microarray dataset are listed (based on chip-Chip analysis), without consideration of statistical significance. For cluster 7 and 8 in Figure 2D, only TFs with T-values < −1 are included.

For calculations of the differences in TF T-values between two different conditions (X-Y), the following rules were set up: If the T-values of the TF has the same sign in both conditions, then purple (positive numbers) indicates a higher absolute value in YPD germination compared to glucose germination/stationary phase exit, and green vice versa (negative numbers). If the TF T-values are of different signs, the highest absolute value of the two conditions dictates the direction of the comparison.

For Cytoscape visualization, the data on transcription factor promoter binding was collected elsewhere [21], and the peak absolute value for each TF T-value in YPD and glucose (combined) was used to set the temporal order. Only those TFs identified by T-profiler with T-values ≥3.5 or ≤ −3.5 (in one or more time points) in one or both conditions compared are included in the analyses.

## Competing interests

The authors declare that they have no competing interests.

## Authors’ contributions

CG participated in the design of the study, analyzed and interpreted the data, made figures, participated in discussions of the work and has been highly involved in drafting the manuscript. IP designed and carried out the microarray analysis and performed initial data analysis. WV and AE performed the statistical analysis and processing of data and helped with initial data analysis and interpretation. JN participated in design and coordination of the study. MK assisted and advised CG on analysis and interpretation of the data and has been highly involved in drafting the manuscript. SH conceived of the study, and participated in its design and coordination and has been involved in drafting the manuscript. All authors read and approved the final manuscript.

## Supplementary Material

Additional file 1 **K-means clustering of the complete gene expression dataset of YPD induced germination is largely consistent with clusters in Figure**1**.** Genes were subjected to K-means clustering and grouped in eight clusters. The graph displays the average expression profiles of the eight clusters, named and coloured according to the clusters in Figure 1B &1C. Non-responsive clusters are collectively numbered 0 and coloured grey. Click here for file

Additional file 2 **T-profiler analysis of the global K-means clusters identified in Additional file****1****.** TFs identified both in the global analysis in Figure 2A and in the eight clusters identified in Additional file 1 are listed. The TFs identified also in Figure 2D are highlighted. In brackets are the numbers of genes in each cluster. Click here for file

Additional file 3 **Two-dimensional visualisation highlights the correlation between the transcriptional responses of spores to rich growth medium and pure glucose.** Two dimensional hierarchical clustering of the expression response to YPD and glucose. The x-axis represents the YPD cluster trees whereas the y-axis holds the glucose cluster trees. Three plots are separated by a grey line; genes whose expression changed two-fold in both data sets to the upper left, genes whose expression changed two-fold after glucose addition but not after YPD addition to the upper right, genes whose expression changed two-fold after YPD addition but not after glucose addition to the lower left. Each cluster is subdivided into groups of genes with correlation factor >0.75 by thin lines. The colour panels along each axis indicate diminished (blue) or stimulated gene expression (red) over time, compared to dormant spores. Each gene occurs once on each axis and the intersection is marked with “■”. Click here for file

Additional file 4 **Two-dimensional comparison of the YPD data set with previous study on yeast germination.** Two dimensional hierarchical clustering of the transcriptional response of spores to YPD, comparing the datasets from this study and a previous study (Joseph-Strauss) [11]. The x-axis represents the “YPD this study” cluster trees whereas the y-axis holds the “YPD Joseph-Strauss” cluster trees. Three plots are separated by a grey line; genes whose expression changed two-fold in both data sets to the upper left, genes whose expression changed two-fold in Joseph-Strauss dataset but not in this study to the upper right, genes whose expression changed two-fold in this study but not in the Joseph-Strauss dataset to the lower left. Each cluster is subdivided into groups of genes with correlation factor >0.75 by thin lines. The colour panels along each axis indicate diminished (blue) or stimulated gene expression (red) over time, compared to dormant spores. Each gene occurs once on each axis and the intersection is marked with “■”. Click here for file
