# Supplementary figures and images for: Time course gene expression profiling of yeast spore germination reveals a network of transcription factors orchestrating the global response
Source: BMC Genomics. 2012 Oct 15;13:554. doi: 10.1186/1471-2164-13-554 (PMC3577491; doi:10.1186/1471-2164-13-554)

## Additional file 1

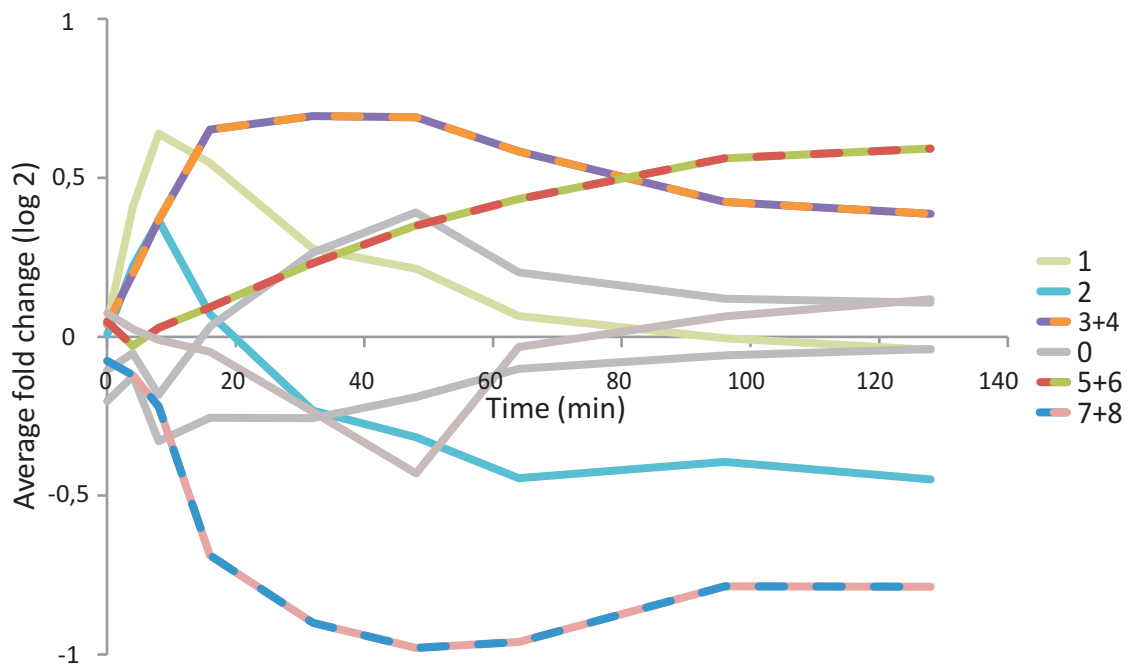

Supplement: Additional file 1 — K-means clustering of the complete gene expression dataset of YPD induced germination is largely consistent with clusters in Figure1. Genes were subjected to K-means clustering and grouped in eight clusters. The graph displays the average expression profiles of the eight clusters, named and coloured according to the clusters in Figure 1B &1C. Non-responsive clusters are collectively numbered 0 and coloured grey. [file 1471-2164-13-554-S1.pdf]
