# Supplementary material for: Time course gene expression profiling of yeast spore germination reveals a network of transcription factors orchestrating the global response
Source: BMC Genomics. 2012 Oct 15;13:554. doi: 10.1186/1471-2164-13-554 (PMC3577491; doi:10.1186/1471-2164-13-554)
Supplement: Additional file 2 — T-profiler analysis of the global K-means clusters identified in Additional file1. TFs identified both in the global analysis in Figure 2A and in the eight clusters identified in Additional file 1 are listed. The TFs identified also in Figure 2D are highlighted. In brackets are the numbers of genes in each cluster. [file 1471-2164-13-554-S2.pdf]

Additional file 2

**Cluster 1** (569)		**Cluster 2** (476)		**Cluster 3+4** (1268)		**Cluster 5+6** (756)		**Cluster 7+8** (1749)	
TF	T-value	TF	T-value	TF	T-value	TF	T-value	TF	T-value
HSF1	5.08	NRG1	3.26	YAP6	3.57	STE12	9.47	PHD1	3.16
PHD1	3.17	HSF1	3.04	CBF1	3.27	FHL1	8.99	YAP6	2.85
MSN4	3.01	SOK2	1.83	RTG3	1.47	RAP1	6.67	NRG1	2.37
MSN2	2.47	FHL1	1.77	NRG1	1.45	DIG1	4.85	FHL1	1.56
NRG1	2.42	PHD1	1.64	UME6	1.41	SFP1	3.74	STE12	1.35
YAP6	1.90	NRG1	1.42	HSF1	1.29	PHD1	2.70	ROX1	1.27
CBF1	1.42	UME6	1.31	PHD1	1.12	NRG1	2.01	ABF1	1.07
UME6	1.08	MSN2	1.28	MSN2	0.88	YAP6	1.96	DIG1	0.97
SOK2	1.05	YAP6	0.75	RAP1	0.51	HAP1	1.21	SOK2	0.93
FHL1	1.03	RAP1	0.44	HAP1	0.32	ROX1	0.73	CBF1	0.59
STE12	0.89	HAP1	-0.02	ROX1	0.23	ABF1	-0.57	RAP1	0.50
RAP1	0.12	RTG3	-0.45	ABF1	-0.32	UME6	-0.75	HAP1	-0.57
HAP1	-0.64	CBF1	-0.54	SFP1	-0.59	CBF1	-1.00	UME6	-1.48
ABF1	-0.64	MSN4	-0.98	DIG1	-1.09			RTG3	-1.56
		STE12	-1.14	MSN4	-1.30			MSN4	-3.39
		ABF1	-2.25	STE12	-1.48			HSF1	-3.52
				FHL1	-2.27			MSN2	-4.47
								GCR2	-4.94
